# Polychlorinated Biphenyls (PCBs) Enhance Metastatic Properties of Breast Cancer Cells by Activating Rho-Associated Kinase (ROCK)

**DOI:** 10.1371/journal.pone.0011272

**Published:** 2010-06-23

**Authors:** Sijin Liu, Shitao Li, Yuguo Du

**Affiliations:** 1 State Key Laboratory of Environmental Chemistry and Ecotoxicology, Research Center for Eco-Environmental Sciences, Chinese Academy of Sciences, Beijing, China; 2 Department of Pathology, Harvard Medical School, Boston, Massachusetts, United States of America; Health Canada, Canada

## Abstract

**Background:**

Polychlorinated biphenyls (PCBs) are a family of structurally related chlorinated aromatic hydrocarbons. Numerous studies have documented a wide spectrum of biological effects of PCBs on human health, such as immunotoxicity, neurotoxocity, estrogenic or antiestrogenic activity, and carcinogensis. The role of PCBs as etiologic agents for breast cancer has been intensively explored in a variety of *in vivo*, animal and epidemiologic studies. A number of investigations indicated that higher levels of PCBs in mammary tissues or sera correlated to breast cancer risk, and PCBs might be implicated in advancing breast cancer progression.

**Methodology/Principal Findings:**

In the current study, we for the first time report that PCBs greatly promote the ROCK activity and therefore increase cell motility for both non-metastatic and metastatic human breast cancer cells *in vitro*. In the *in vivo* study, PCBs significantly advance disease progression, leading to enhanced capability of metastatic breast cancer cells to metastasize to bone, lung and liver. Additionally, PCBs robustly induce the production of intracellular reactive oxygen species (ROS) in breast cancer cells; ROS mechanistically elevate ROCK activity.

**Conclusions/Significance:**

PCBs enhance the metastatic propensity of breast cancer cells by activating the ROCK signaling, which is dependent on ROS induced by PCBs. Inhibition of ROCK may stand for a unique way to restrain metastases in breast cancer upon PCB exposure.

## Introduction

Polychlorinated biphenyls (PCBs) are chlorinated aromatic hydrocarbons with 209 congeners. They are highly stable and lipophilic chemicals widely distributed in the environment. Although the production and massive use of PCBs have been banned in the 1970 s, they persist in the environment, biomagnify through food chains and consequently accumulate in fat tissues in humans [1], [2], [3]. For many years, PCBs are concerned as etiologic agents for breast cancer and other cancers. Initial studies indicated higher levels of PCBs in mammary tissues or sera corresponded to increased risk of breast cancer [4], [5]; however, later studies showed no positive association between PCB exposure and breast cancer development, with possible positive correlations to some specific PCB congeners or among particular populations [6], [7], [8]. Some recent investigations suggested that PCBs were implicated in promoting breast cancer progression, and some particular PCB congeners might contribute to high-grade tumors and overall poor prognosis in breast cancer patients [9], [10], [11].

Breast cancer is the most commonly diagnosed cancer and the second leading cause of cancer-related deaths among women. The real threat to lives of breast cancer patients is not the primary tumor in the breast, but secondary tumors in other organs (*e.g.* bone and lung) which accounts for 90% of deaths of breast cancer patients. Once breast cancer cells metastasize and form tumors in distant organs, the disease is incurable with available therapeutics [12]. Until now, the underlying mechanisms responsible for breast cancer metastasis remain to be elucidated, and there is by far no metastasis-specific therapy in clinical practice.

The Rho-associated kinases, ROCK 1 and 2 (here referred to as ROCK), are major mediators of Rho activity [13]. ROCK is implicated in the regulation of *in vitro* invasion and motility and *in vivo* metastasis of cancers [14]. Clinical studies show that the expression of ROCK is significantly increased in human breast cancer tumors with metastases than those without metastases [15], [16]. And increased expression of ROCK is associated with higher pathological grades and later stages, and its expression level is strongly correlated to the overall survival in breast cancer patients [15]. Overexpression of ROCK can significantly enhance *in vitro* cell invasion/migration in cancer cells [17], [18], [19]; however, the expression of the dominant-negative ROCK and the ROCK inhibitor, Y27632, can massively suppress *in vitro* cancer cell invasion/migration and *in vivo* motility and dissemination [18], [20], [21], [22].

In this study, **t**o delineate the associations between the PCB exposure and breast cancer risk and progression, we assess the effects of PCBs on the tumorigenic and metastatic features of breast cancer cells. Overall, we describe a novel role of PCBs in enhancing metastatic properties of breast cancer cells by activating the ROCK signaling.

## Results and Discussion

**Figure 1:**
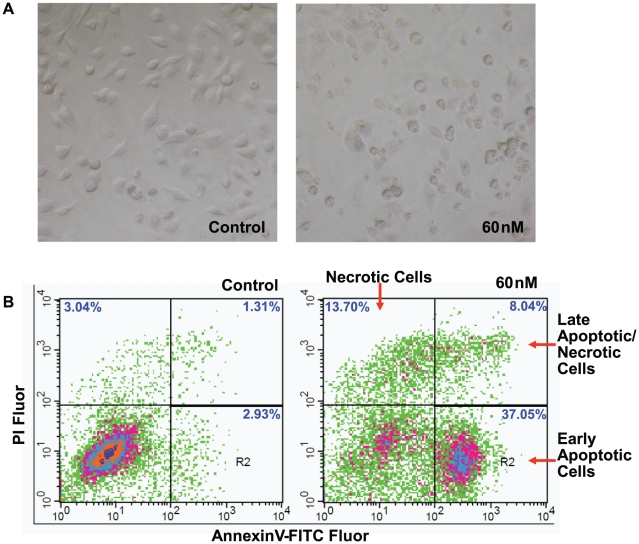
The cytotoxicity induced by PCBs on MDA-MB-231 cells. (A) Phase-contrast images of cell morphology from MDA-MB-231 cells treated with/without the PCB mix at 60 nM for 24 hrs. Original maginification, ×200. (B) FACS analysis of apoptosis induced by PCBs at 60 nM for 24 hrs in MDA-MB-231 cells using FITC-Annexin V and PI stains. Early apoptotic cells (lower right), necrotic cells (upper left) and late apoptotic/necrotic cells (upper right) are shown as arrows indicate.

The concerns with respect to likely carcinogenic influence on mammary tissues with exposure to PCBs derived from the estrogenic activity of these compounds, which have the ability to mimic or interfere with the action of endogenous hormones [23], [24], [25]. Because of their lipophilic nature, PCBs tend to accumulate in adipose tissues, and therefore could be detectable in human mammary tissues and even in milk. Epidemiological studies suggested a number of congeners are simultaneously detected in serum, breast milk and adipose tissue in humans [6], [8], [26], [27]. Due to their estrogenic activity, PCBs have been documented to disrupt the endocrine systems of animals and humans [28], [29], and the combination of different PCBs could behave synergistically in presenting their estrogenic potency [30]. To mimic co-exposure to PCB congeners, in this study we used the commercially available PCB mix (equal amount of No. 28, 52, 101, 138, 153, 180 and 209) of frequently detected PCBs in human subjects. We employed estrogen receptor-positive (ER^+^) MCF-7 cells and estrogen receptor-negative (ER^-^) MDA-MB-231 cells in order to discriminate the ER-dependent and -independent effects. There was a rather mild effect of the PCB mix on cell growth and survival at low concentrations (<60 nM) for both ER^+^ MCF-7 and ER^-^ MDA-MB-231 cells (data not shown); however, PCBs exerted significant cytotoxicity on these cells and induce cell death at high concentrations (>60 nM). As shown in Fig. 1A, distinct apoptotic morphology was observed in MDA-MB-231 cells treated with 60 nM PCBs for 24 hrs compared to the vehicle control, as the PCB-treated cells became round and tended to detach from the plate. The apoptosis induced by PCBs was confirmed by an analysis of flow cytometry using FITC-Annexin V and propidium iodide (PI) stains. PCBs dramatically induced cell death as indicated by increased early apoptotic cells (37.05% VS 2.93%), necrotic cells (13.70% VS 3.04%) and late apoptotic/necrotic cells (8.04% VS 1.31%) (P<0.001, n = 3), compared to the control (Fig. 1B).

**Figure 2:**
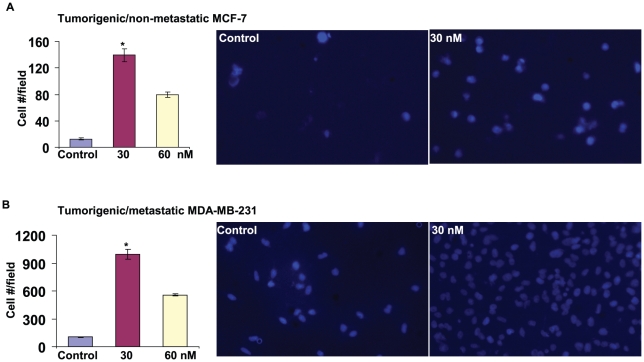
PCBs enhance cell migration in breast cancer cells. Cell motility was examined from a transwell migration assay in MCF-7 and MDA-MB-231 cells treated with the PCB mix at 30 or 60 nM for 24 hrs. After the DAPI staining (blue), two images were randomly taken from three individual replicates under a microscope, and transmigrated cells in the chamber filters in each image were counted. Representative images for MCF-7 (A) and MDA-MB-231 (B) cells are shown, and the numbers of transmigrated cells were quantified (n = 6). *, P<0.001, compared with the vehicle control and the 60 nM group.

The previous studies indicated that PCBs were correlated with more aggressive breast cancers, and might promote tumor progression [9], [10], [11]. Thus, the cell motility influenced by the PCB mix was assessed in a transwell migration assay as previously described [16]. Compared to the non-metastatic cells, MCF-7, metastatic cells, MDA-MB-231, displayed robust migration ability, as the number of transmigrated cells was 8 times greater than that of MCF-7 cells (P<0.001, n = 6) (Fig. 2A&B). Upon the stimulation of PCBs at 30 nM for 24 hrs, the cell motility was tremendously increased (∼10 times) for both ER^+^ MCF-7 and ER^-^ MDA-MN-231 (Fig. 2A&B, P<0.001), suggesting the presence of ER is not an indispensable for PCB-facilitated cell migration. At higher concentrations of PCBs (e.g. 60 nM), the cell motility was greatly reduced (Fig. 2A&B, P<0.001) presumably due to their cytotoxicity as discussed above (Fig. 1). In addition, all PCB congeners (namely 28, 52, 101, 138, 153, 180 and 209) used in this study are not structurally similar to 2,3,7,8-tetrachlorodibenzo-*p*-dioxin (TCDD) which has the ability to bind to and activate the ligand-activated transcription factor, the aryl hydrocarbon receptor (AhR). These non-dioxin-like PCBs therefore have no ability to bind to the AhR and activate its downstream signaling [31]. Thus, the PCB-promoted effect on cell motility is independent of the ER signaling and the AhR signaling as well.

**Figure 3:**
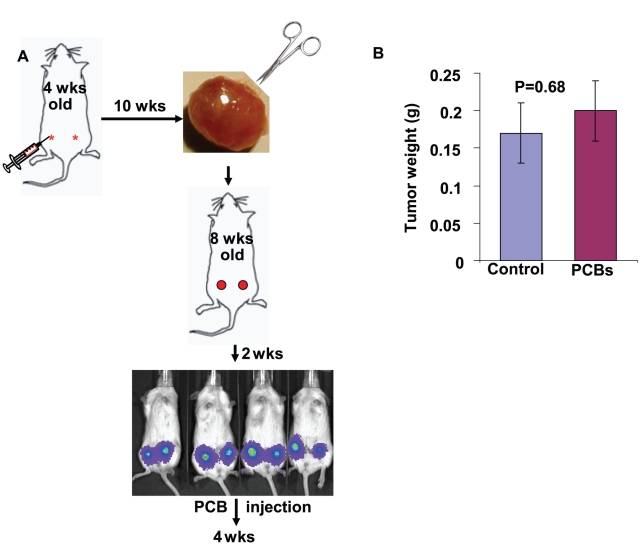
Role of PCBs in breast cancer progression and metastasis in the mouse model. (A) A schematic of the mouse model used in this study. The details about the model are described in the *Methods*. (B) The average weight of primary tumors in the PCB-treated mice and the vehicle control mice (n = 8).

**Figure 4:**
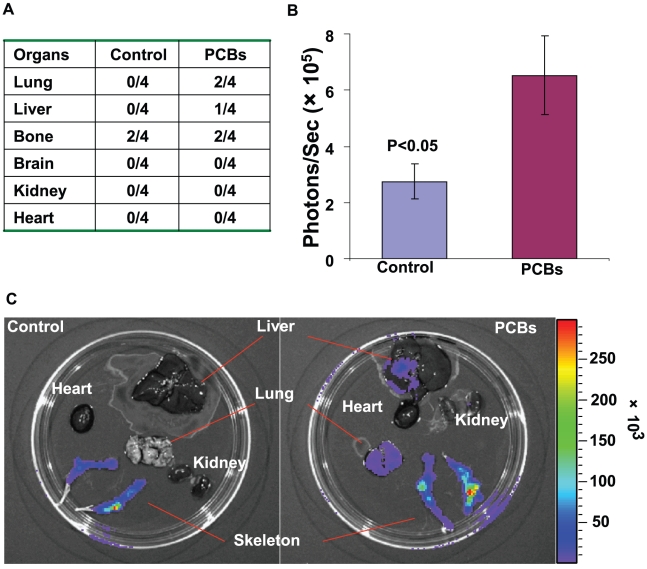
PCBs enhance MDA-MB-231 breast cancer cell metastases *in vivo*. (A) The occurrence of metastases in all organs tested in the PCB-treated mice and vehicle control mice. Metastases were examined using the Xenogen 2000 and the IVIS software as previously described [16]. (B) The quantified data of metastatic tumors (reflected by photon flux, photons/sec [16]) in mouse skeleton (n = 4). (C) Representative images of metastases in mouse liver, lung and skeleton from the bioluminescent imaging.

To gain insight into the role of PCBs in regulating cell migration/invasion, we looked into the changes of metastatic capability of MDA-MB-231 cells upon exposure of PCBs *in vivo* using a mouse model of breast cancer metastasis (Fig. 3A). As shown in Fig. 3B, the growth of primary tumors was little affected after 4 weeks of PCB exposure, consistent with the *in vitro* observation on cell proliferation (data not shown). However, PCBs greatly enhanced the capability of MDA-MB-231 cells to metastasize to lung, liver and bone. 2 out of 4 mice developed lung metastasis, and 1 out of 4 mice developed liver metastasis among the PCB-treated mice. In contrast, no mice in the vehicle control group developed either lung or liver metastasis (Fig. 4A&C). Although 50% of mice for both groups developed bone metastasis in bilateral hind limbs, the mass of metastatic tumors in the PCB-treated mice was significantly greater than that in the control mice (Fig. 4B&C, n = 4, P<0.05). This observation is consistent with a previous epidemiological finding that high concentrations of PCBs in mammary adipose tissues were associated with high-grade tumors and overall poor prognosis in breast cancer patients [11].

**Figure 5:**
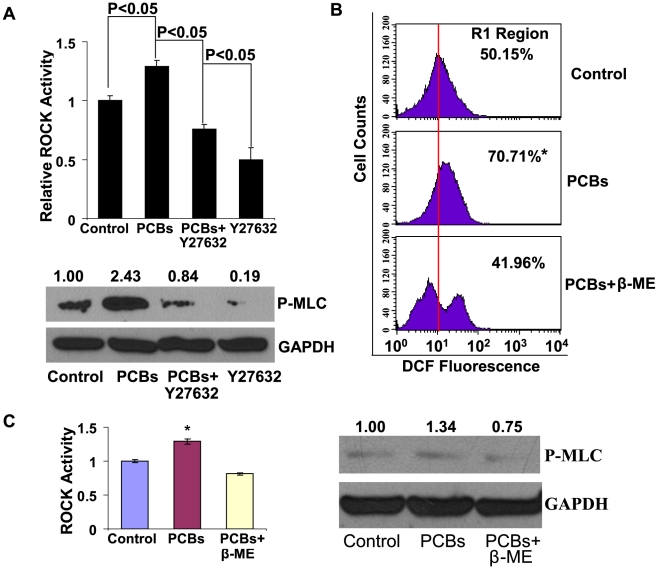
The signaling stimulated by PCBs in MDA-MB-231 cells. (A) The relative ROCK activity and Western blot analysis of P-MLC in MDA-MB-231 cells treated with the PCB mix (30 nM) with or without the ROCK inhibitor, Y27632 (10 µM) for 24 hrs (n = 3). (B) ROS production in MDA-MB-231 cells upon PCB treatment. DCF fluorescence in cells were measured by FACS analysis after 6-hr PCB treatment with or without β-ME (14.3 µM) (n = 3). (C) The relative ROCK activity and Western blot analysis of P-MLC in cells upon PCB treatment for 6 hrs with or without β-ME (n = 3). *, P<0.05, compared with the vehicle control and the PCBs+β-ME group. The intensities of autoradiogram in Western blots were quantified with Image J (rsbweb.nih.gov/ij). The quantified data for P-MLC were normalized to those of GAPDH.

To shed light on the mechanism by which PCBs promote *in vitro* cell motility and *in vivo* metastasis of breast cancer cells, we investigated the potential downstream targets of PCBs. Illuminated by the increased cell motility, we assessed the effect of PCBs on the ROCK activity. PCBs significantly activated ROCK activity by 30% or so, and this effect could be attenuated by a specific ROCK inhibitor, Y27632(Fig. 5A, n = 3, P<0.05). Corresponding to the changes of ROCK activity, the substrate of ROCK, myosin light chain (MLC), was more phosphorylated upon the treatment of PCBs than the vehicle control; the increase of phosphorylated MLC (P-MLC) was weakened by Y27632 (Fig. 5A). PCBs largely induced the production of intracellular reactive oxygen species (ROS) in breast cancer cells by 40% (Fig. 5B), along with the increase of ROCK activity and the level of phosphorylated MLC (Fig. 5C). Importantly, we demonstrated that the increase of ROS mechanistically led to elevated ROCK activity induced by PCBs, as the increase for both the ROCK activity and the level of phosphorylated MLC could be undermined by the pre-treatment of beta-mercaptoethanol (β-ME) (Fig. 5C, n = 3, P<0.05).

Although the ability of PCBs in provoking tumor initiation and development has been observed, the mechanism remains to be elucidated. Numerous possible mechanisms have been explored, such as the estrogenic activity, effects on vitamin A metabolism and intercellular communication, and induction of oxidative stress (ROS) [32], [33].The potential interaction between ROCK- and ROS-signal transduction pathways has been suggested in previous studies [34], [35], [36]: ROCK is activated by arsenic trioxide (As_2_O_3_), a strong ROS-inducer [35]. Further, hypoxia-induced ROS activates the RhoA/ROCK pathway [36].

**Figure 6:**
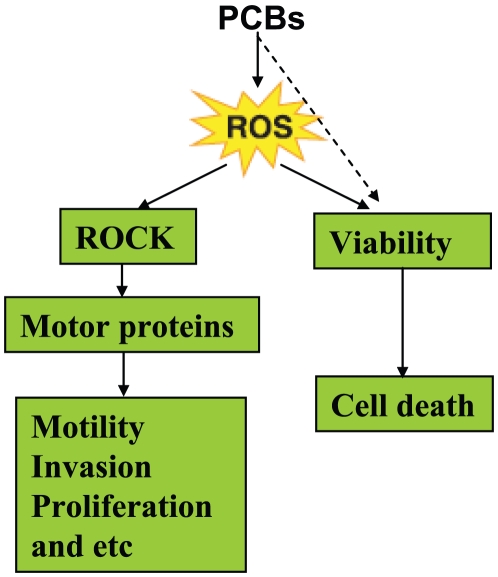
A schematic of PCB-induced signaling in breast cancer cells. At low concentrations, PCBs activate ROCK kinase activity to regulate the actin-myosin-dependent contraction by phosphorylating motor proteins, such as the regulatory MLC. The resulting effect of ROCK activation leads to increased cell motility and potentially metastasis. At high concentrations, PCBs cause cell death via apoptosis, which may be dependent on ROS or not.

PCBs are widespread and persistent environmental pollutants that impose potential hazards on human health. The possible correlation between environmental PCB exposure and breast cancer risk exists biologically plausible, and mounting evidence indicates that PCB exposure contributes to the aggressiveness and metastases of breast cancer. The findings from the current study suggest that PCBs potently augment the ROCK signaling to advance breast cancer metastases, and the activation of ROCK by PCBs is dependent on ROS, but independent of the ER signaling and the AhR signaling (Fig. 6). Inhibition of ROCK, therefore, appears to represent a novel therapeutic approach for metastases in breast cancer upon PCB exposure.

## Materials and Methods

### Chemicals

The PCB congener mix (equal amount of No. 28, 52, 101, 138, 153, 180 and 209) was purchased from Sigma. Stock solutions of these compounds were diluted in 100% ethanol and added to the culture medium immediately before use. Equal volume of PBS corresponding to different concentrations of the PCB mix was diluted in 100% ethanol as the vehicle control.

### Human Breast cancer cell lines and cell culture

Human Breast cancer cell lines, MCF-7 and MDA-MB-231 were stored and cultured as described previously [16], [37], [38].

### Animal experiments

All mouse care and experimentation were approved by the Committee of Animal Care at the RCEES, Chinese Academy of Sciences. Immunodeficient (NOD/SCID) mice were maintained under aseptic sterile conditions. Surgeries were performed under sterile conditions and mice received antibiotics (trimethoprim sulfa) in the drinking water for 2 weeks following all surgical procedures. Experimental set-up was similar to the methods described in our previous publication [16]. Briefly, bilateral fourth mammary fat pads (MFPs) were injected with 1 million MDA-MB-231 breast cancer cells after a midline incision was made and the fourth MFP was visualized on each side of the mouse. Cells were injected in 1∶3 diluted 20 µl matrigel (BD Biosciences):sterile PBS using a Hamilton syringe (Hamilton Co.). 10 wks later when the tumor size reached about 200 mm^3^, the mouse was sacrificed. The primary tumors were removed and cut into small pieces in a cube shape with a scissor. 0.02 g of tumor tissues were implanted underneath MFPs of 8 wk old mice. 2 weeks later when primary tumors reached ∼75 mm^3^, one group of mice received one dose of intraperitoneal (i.p.) injection of the PCB mix diluted in ethanol (3 nM/kg, in 100 µl).Control mice received ethanol-diluted PBS.

Bioluminescent imaging was performed 4 wks post the injection, with both primary tumors and metastases examined using the Xenogen 2000 and the IVIS software. Mice were administrated i.p. injections of luciferin (100 nl of 1 mg/mL; Molecular Probes), and after 10 minutes, anesthetized (isoflurane inhalation) for imaging of primary tumors. Following euthanization using CO_2_, mouse hind limbs, lungs, livers, kidneys and other tissues were removed for separate imaging, and detection of metastases.

### Western blot analysis

Protein extracts were prepared from cells, and 20 µg of each was used for separation by 4–12% SDS-PAGE and processed for Western blot analyses as described previously [39]. Antibodies used were the anti-GAPDH (1∶1,000, Santa Cruz Biotechnology), and anti–phosphorylated MLC (1∶1,000, Sigma).

### FACS analysis

Apoptosis analysis was analyzed using FITC-conjugated Annexin V and PI as described previously [40]. The level of cellular ROS was examined by FACS analysis using cells treated with 5 µM of DCF (Molecular Probes; Invitrogen) at 37°C as previously described [39]. Briefly, cells were incubated with DCF in DMEM medium for 30 minutes and then treated with PCBs (60 nM) with or without β-ME (14.3 µM) for 6 hrs. Cells were then collected for analyses.

### ROCK kinase activity assay

Breast cancer cells were treated with PCBs in medium with 1% FBS for 24 hrs at 37°C and 5% CO_2_. After two cold PBS washes, 1×10^6^ cells were lysed with M-PER Mammalian Protein Extraction Reagent (Pierce). ROCK kinase activity was determined by the Rho-kinase Assay kit (Cyclex Co, Japan) according to the manufacturer's protocol. The kinase activity in the vehicle control was defined as 1.

### Transwell migration assay

After breast cancer cells were serum-starved for 24 hrs, cell motility was assessed by the transwell (Corning) migration assay following the standard protocol. Cells were plated on the upper chamber (1×10^4^/insert) in 1% serum medium with or without PCBs and allowed to migrate for 24 hrs with 10% serum medium in the bottom as chemoattractant. Cells on the filter-side of the upper chamber were cleaned with cotton swabs and the filters were stained with 1×DAPI solution after cold methanol fix. Cell nuclei on the filters were visualized under a fluorescent microscope.

### Statistical analysis

Two-tailed Student's t-test was used to analyze the statistical significance of experimental data. All results are presented as mean±SEM, and a P-value of <0.05 determined statistical significance.
